# Important and Feasible Actions to Address Cervical Screening Participation amongst South Asian Women in Ontario: A Concept Mapping Study with Service Users and Service Providers

**DOI:** 10.3390/curroncol31070301

**Published:** 2024-07-17

**Authors:** Kimberly Devotta, Patricia O’Campo, Jacqueline Bender, Aisha K. Lofters

**Affiliations:** 1 Dalla Lana School of Public Health, University of Toronto, Toronto, ON M5T3M7, Canada; 2 Women’s College Hospital, Toronto, ON M5S1B2, Canada; 3 St. Michael’s Hospital, Unity Health Toronto, Toronto, ON M5B1W8, Canada; 4 University Health Network, Toronto, ON M5G2C4, Canada; 5 Department of Family and Community Medicine, University of Toronto, Toronto, ON M5G1V7, Canada

**Keywords:** cervical cancer, cervical screening, community engagement, concept mapping, cervical screening interventions

## Abstract

Regular cervical screening can largely prevent the development of cervical cancer and innovative methods are needed to better engage people in screening. In Ontario, Canada, South Asian women have some of the lowest rates of screening in the province. In this study, we used concept mapping to engage two stakeholder groups—South Asian service users and service providers—to identify and prioritize points of intervention to encourage the uptake of cervical screening. After participants brainstormed a master list of statements, 45 participants rated the statements based off ‘importance’ and ‘ease to address’ in relation to encouraging cervical screening. A bivariate plot (X-Y graph) that shows the average rating values for each statement across the two rating variables (a ‘go-zone’ display) was produced to display priorities for implementation. Statements that were considered high priority to address reflected issues around education and awareness including understanding and communication related to cervical screening and preventative care, as well as the need for trusted sources of information. Statements that were considered high priority but challenging to implement were centered around fear, stigma, discomfort, family and personal priorities. This study highlighted that stigma, norms and social relations that impact the uptake of screening must be addressed in order for education and awareness raising to be effective and to move people from conviction around screening to action.

## 1. Introduction

Regular screening is effective in preventing the development of almost all cases of cervical cancer. With a long precancerous stage, accurate and timely cervical screening can find and treat abnormal cells before they develop into cancer, emphasizing the importance of regular cervical cancer screening for people with a cervix. To support this, many jurisdictions in Canada and around the world have developed organized screening programs. In Ontario, Canada, the Ontario Cervical Screening Program (OCSP) was established in the year 2000. Its introduction saw an initial increase in cervical cancer screening rates, but these rates have remained stable at around 60% since 2013, well below provincial targets of 85% [1]. Furthermore, cervical cancer screening rates are projected to have been greatly impacted by the COVID-19 pandemic, with the province working through a large backlog of missed screenings due to public health measures that impacted the availability and delivery of Pap tests [2].

The World Health Organization (WHO) and the Canadian Partnership Against Cancer (CPAC) have recently set out to accelerate the elimination of cervical cancer, and a main part of this strategy is through increased screening participation and a switch from Pap testing to HPV testing that is forthcoming in Ontario [3,4]. Strengthening the current cervical screening landscape in Ontario, while also looking toward supporting a major change and goal for cervical screening and cancer, calls for innovative and timely research. Currently, the OCSP recommends that everyone with a cervix who has been sexually active should commence cytology-based screening at the age of 25—at the time of this study, it was 21 years—with some exceptions (e.g., those who are immunocompromised). The OCSP has a systematic invitation and recall component to encourage screening uptake [5]. However, certain groups of women remain underscreened. Immigrants and people living with a low income—those at or below Statistics Canada’s low-income cutoff—have some of the lowest cervical screening participation rates in Ontario [6,7]. South Asian women, in particular, have the lowest rates of screening [7,8]. Data from the 2017 Canadian Community Health Survey found that immigrant women had self-reported lower adherence to cervical screening than their Canadian-born counterparts, with sub-group analyses finding that South Asian women were the least likely to report being adherent [9].

Studies throughout Canada and internationally have sought to identify the barriers that cause women to delay or not participate in cervical screening. Being asymptomatic or not understanding preventative care have been well documented as barriers in cervical screening, and particularly so for South Asian women [10,11,12,13,14,15,16]. Discomfort with Pap tests is often cited [14,17,18,19]. Misconceptions such as believing that Pap tests are for sexually transmitted infections or that healthy lifestyles prevent cancer can cause women to delay or avoid screening [20,21]. Knowledge about where to get screened and having the time to do so are often cited as barriers [15,22]. In some cases, moving from a ‘curative health care system’ in their country of origin to a preventative health system such as Canada can pose a significant knowledge barrier to screening, as they are not used to viewing health in this new lens [11].

Work is still needed to develop culturally informed interventions that are both effective and sustainable for addressing underscreening [23,24]. What are needed are participant-driven methods for identifying and prioritizing actions to address cervical screening amongst South Asian women. There are many factors that impact cervical screening participation amongst South Asian women that extend beyond healthcare settings. While many studies have identified trends in screening and barriers to screening, the current study seeks to elicit ideas and priorities for action directly from those impacted (South Asian services users, and people who work with and serve South Asian people in healthcare services and the community). We engaged South Asian women eligible for screening in Ontario and their service providers (e.g., healthcare providers, health promoters, community champions, settlement workers) in a concept mapping study to understand the following: how do the lives and experiences of South Asian women shape their decisions around getting screened for cervical cancer? Concept mapping (CM) was chosen because it engages multiple stakeholders to identify issues that need to be addressed and to also develop reasonable solutions [25,26]. Using concept mapping, we asked participants to identify high-priority ideas for interventions and investigated other important and challenging areas of intervention implementation. In this paper, we present community-identified strategies for addressing and improving cervical screening participation amongst South Asian women.

## 2. Materials and Methods

### 2.1. Research Ethics

This study was approved by the Research Ethics Board of the University of Toronto (REB# 43281).

### 2.2. South Asian Ethnicity

Race/ethnicity is a social construct that can have many impacts, including creating unequal power social relations in healthcare [27]. This unequal power can greatly affect how or even if people access healthcare and the quality of care they receive. It can also impact who has historically had input in the design and delivery of services and whose voices are heard today. This can create new, or worsen existing, health inequities. As such, engaging members of the South Asian community in identifying and prioritizing areas of intervention can make a critical difference in addressing underscreening as it centers their experiences and opinions. In this study, we define ‘South Asian’ as ancestry and not necessarily place of birth, meaning the term was inclusive of those who immigrated to Canada from South Asian countries, as well as those who were born in Canada or non-South Asian countries but could trace their origins back to South Asian countries (e.g., Indo-Guyanese people). South Asian ancestry can include ancestors from the following countries: Afghanistan, Bangladesh, Bhutan, India, Maldives, Nepal, Pakistan and Sri Lanka.

### 2.3. Study Design

A concept mapping (CM) study design was chosen to understand how different aspects of South Asian women’s lives and experiences impact cervical screening. This method brings together qualitative approaches and quantitative analytical tools to facilitate a group of diverse stakeholders to articulate their ideas and then represent them in quantitatively derived visual results [25,26]. Given the need to engage South Asian women alongside providers in healthcare and community services to identify and prioritize actions, CM was chosen for its collaborative nature and many participatory aspects.

CM is composed of a series of participatory activities that can each be used to understand how a group thinks about an issue. After brainstorming the items for the master list, two conceptual activities are performed to structure the statements in the conceptual domain: sorting and rating. In the sorting activity, participants independently review all of the statements and sort them into piles that make sense to them and reflect how they think the statements are related to each other. The concept maps are produced from these data. The rating activity asks participants to independently rate the statements on one or more rating questions that seek to measure a particular view (e.g., importance, feasibility). This is used to create a go-zone graph (a comparison of average rating values for two different variables) that we present in this paper. The concluding activity is map interpretation, where a group of participants are brought together to review and comment on the concept mapping activity analyses [26,28]. 

We used GroupWisdom (Version 2013.322.11), a concept mapping software, to manage and analyze the data.

### 2.4. Participant Sampling and Recruitment

Non-random sampling is used in CM to gather a broad set of ideas [25]. In this study, a purposive sampling strategy was used for heterogeneity in the participant sample, which consisted of the following: (i) South Asian women living in the Greater Toronto Area (GTA), (ii) community champions—trusted female members of the South Asian community with pre-existing connections with local community groups and organizations, (iii) people who work in organizations that serve South Asian women in the GTA and (iv) healthcare providers serving South Asian patients. Working with a community champion, participants were recruited through word-of-mouth and poster distribution on social media and services within communities with a large South Asian population. We also distributed our poster via email distribution lists within hospitals, primary care teams and services within the community. Participants were not obligated to participate in each CM activity. As the activities progressed, additional participants were recruited.

All participants had to be at least 18 years of age and speak conversational English. The eligibility criteria for South Asian women included self-identify as South Asian, and is or has ever been eligible for cervical screening in Ontario (at least 21 years of age, has been sexually active, has a cervix). Participants from healthcare services and the community had to identify as being in a role that works or serves South Asian women. This included healthcare providers, social service workers, health promoters, community organizers and others in the healthcare and social services. Additionally, they had to answer questions that gauged their familiarity with cervical cancer screening amongst South Asian women. 

### 2.5. Demographics

We collected demographics before the start of each CM activity. Participants categorized themselves into one of the following 5 categories that they felt best described them: (i) I identify as South Asian; (ii) I work in a role or an organization that serves South Asian women and I identify as South Asian; (iii) I work in a role or an organization that serves South Asian women and I do not identify as South Asian; (iv) I work as a primary care provider and I identify as South Asian; or (v) I work as a primary care provider and I do not identify as South Asian. All participants were asked their age and gender identity. Service providers (i.e., categories ii to v) were asked how long they have been in their area of work, and service users (i.e., category i) were asked if they had ever had a Pap test. Additional demographic questions for service providers were added after the brainstorming activity for the sorting and rating activities. These additional demographic questions asked about the approximate percentage of South Asian people that they serve and further details about their roles in healthcare services and the community.

### 2.6. Concept Mapping Data Collection Activities

Data collection took place from September 2022 to August 2023. The map interpretation activity was completed over Zoom, while the rest of the activities were completed either directly through the GroupWisdom platform or during a group event using a paper and pencil, to be later inputted into GroupWisdom. Participants were given an e-gift card upon completion of each activity: CAD 30 for brainstorming, CAD 40 for sorting and CAD 30 for map interpretation.

#### 2.6.1. Identifying Barriers to and Facilitators of Cervical Screening

A more detailed description of the brainstorming activity has been reported elsewhere [29]. In brief, participants independently provided up to 10 responses to the following focal prompt: ‘One thing about the lives and experiences of South Asian women that influence their decision, in a positive or negative way, to get screened (i.e., a Pap test or HPV test) for cervical cancer is…’. In the activity instructions, we asked people to think about South Asian women in their personal and professional lives. To make participation low-barrier and to include as many eligible people as we could, we used an open and anonymous weblink where participants could complete the demographics and brainstorming activity. Once the activity closed, members of the research team reviewed and reduced the brainstormed statements to create the master list of statements about barriers to and facilitators of cervical screening for South Asian women.

#### 2.6.2. Rating Activity to Understand How Participants Perceive the Importance and Ease of Addressing the Brainstormed Items to Encourage Cervical Screening

During the rating activity, participants rated each statement in the master list based on two rating questions that each used a Likert scale. The first question asked participants the following: how much do you agree or disagree that removing or fixing this barrier would improve cervical cancer screening participation amongst South Asian women? A 5-point Likert scale from the value of 1 being ‘strongly disagree’ to 5 being ‘strongly agree’ was used. The second question, ‘how easy do you think it is to solve or address this issue so that South Asian women will be encouraged to participate in cervical cancer screening?’, was asked along with a 5-point Likert scale that ranged from 1 being ‘very difficult to solve or address’ to 5 being ‘very easy to solve or address’. Throughout this paper, we refer to the first question as our rating of ‘importance’ and the second question as our rating of ‘easy to address.’ In CM, all statements are considered important and, to some degree, possible to address. Rating allows us to see what statements people think are particularly important and easy to address.

The rating activity was relatively more time-consuming and complex than the brainstorming activity, so we created login information for participants to complete this activity at their own pace, allowing them to save their work and log back in as many times as they needed. We also held an in-person event for participants to complete this activity on paper. 

### 2.7. Data Analysis

The rating data were analyzed using a go-zone display which is a bivariate plot (X-Y graph) that shows the average rating values for each statement across the two rating variables [25]. The *x*-axis shows the average rating values of one rating question and the *y*-axis shows the average rating values of the second rating question. The scales displayed on each axis represent the full range of ratings. A vertical line is drawn from the mean of the values on the *x*-axis and a horizontal line is drawn from the mean of the values on the *y*-axis. This divides the display into quadrants, with the axes crossing at the overall mean value for each rating variable [25]. A correlation value is also calculated for the graph to show the degree of predictability in the relationship between the two rating variables represented by the graph. 

The go-zone displays participant perceptions of how realistic it would be to implement a particular statement and the impact it would have on the issue. Each statement is placed in a position in one of the four quadrants. The upper-right quadrant—statements above the mean for each axis—represents the items considered most actionable and of higher implementation priority. This is the ‘go-zone.’ In our study, we placed our ‘importance’ rating question on the *x*-axis and our ‘easy to address’ rating question on the *y*-axis. This means the lower-right quadrant has statements with a high importance rating but a lower easy to address rating, indicating that these would be important but challenging to implement [25]. The upper-right (‘go-zone’) and lower-right quadrants are the most important for planning and implementation because they tell us what participants thought were the most important statements to address and their thoughts on feasibility. Comparing the two rating variables, importance and ease of addressing, in the go-zone display is a way to capture participants’ voices on emotional ‘voting’ (importance) and also their ideas about feasibility [28].

### 2.8. Map Interpretation

During the map interpretation session, participants were presented with many CM outputs from the data analysis, including the average rating values per statement, and the go-zone display comparing the ‘importance’ and ‘easy to address’ rating variables. This session was facilitated by the lead author (KD). Participants were encouraged to look for patterns in the rating values and for areas that showed high or low ratings. When looking at the go-zone display, participants discussed statements within and outside the top-right quadrant and what that might mean for implementation.

## 3. Results

Participant numbers varied between CM activities. Up to 72 participants completed the brainstorming activity [29]. The exact number of study participants is unknown, because the weblink to participate was open and anonymous and participants could access the link multiple times to add more statements, each time having to complete the demographic questions again. Twenty-two participants completed the sorting activity and forty-five completed the rating. The final activity was the map interpretation session, which is a much more qualitative activity, and nine people attended this. Of the nine people, seven of them had participated in all of the activities and two were participants who had been recruited during the sorting and rating rounds, participating in all activities except brainstorming. In our analysis of the sorting data, we removed four sorting datasets due to the use of catchall piles, leaving statements by themselves, sorting less than 75% of the statements and sorting statements more than once. For each rating question, we excluded one participant who had used the same rating value for all of their responses. Therefore, our analysis was based on 18 sorting participants and 44 rating participants. 

A detailed breakdown of our collected demographics, by activity, is summarized in Table 1a,b. Overall, almost all of the participants across the four main activities identified as South Asian (one participant in the sorting round and three participants in the rating round did not identify as South Asian), and apart from one person in the brainstorming round, everyone identified as female. Most South Asian service users (i.e., people who selected ‘I identify as South Asian’) had previously had a Pap test. Across all of the activities, the age range from 41 to 50 years was the largest sub-group of participants, with some representation in each activity of the ages from 21 to 60 years. Participants who worked in healthcare services or the community said that between 9% and 85% of their client population was South Asian, and there was a range of years in which people had been engaged with their work.

### 3.1. Participants’ Perceptions on the Importance and Ease of Addressing Each of the Statements

Table 2 presents the average values for the importance and easy to address rating questions for each statement from the master list. Overall, participants rated the statements highly for importance, as the average values ranged from 3.3 to 4.4 on a 5-point scale. Comparatively, some of the statements were rated lower for ‘easy to address’, as the range of the average rating values was wider, ranging from 2.3 to 4.2 on a 5-point scale. This tells us that participants generally found the statements to be very important to address, but not always easy to solve.

### 3.2. Identifying Statements That Are High-Priority for Implementation to Encourage Cervical Screening

**Figure 1:**
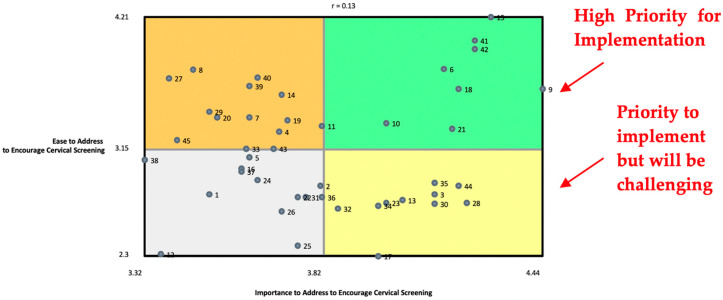
A go-zone graph showing all statements across the two rating variables of ‘importance’ and ‘ease’, where each point is numbered to represent the placement of the corresponding statement (statements and corresponding numbers are detailed in Table 2).

Statements that are high-priority for implementation are those that have, on average, been rated higher than the average ratings for both the ‘important to address’ rating question and the ‘ease to address’ rating question. Figure 1 shows the go-zone graph for importance and ease to address, helping us to see the commonalities and differences in how each statement was rated across the two rating questions. The go-zone in the upper right-hand quadrant shows the statements that are above the mean value for importance (3.82) and ease to address (3.15). The correlation value is 0.13, suggesting no predictable relationship between ratings of importance and ratings for ease to address. This shows us that items that are very important to address are not necessarily easy to address.

The statement ‘Women need reminders to know when they are due for cervical cancer screening’ (#15) was rated the highest for importance and being easy to address, while ‘Men in South Asian households make decisions about females getting screened’ (statement #12) was rated the lowest for importance and being easy to address. Participants in the map interpretation activity were not surprised about statement 12 being rated the lowest, as they believed that men in South Asian households making decisions around cervical screening happens for some, but not very often. They further speculated that if it did not happen often, it can be even more difficult to address for the few women who experience it.

Box 1 presents the statements in the ‘go-zone.’ Many of the statements reflect issues around awareness, understanding and communication related to cervical screening. Ideas specific to cervical cancer and screening, as well as preventative care more generally, are highlighted by these statements. Elements of communication, including needing to communicate with healthcare providers in English, trusted sources of information and the media, are also amongst the statements in this go-zone quadrant that are considered the most impactful and realistic strategies to address and implement. This quadrant emphasizes the need for effective education and information around cervical screening, including how and who is delivering it.

Box 1 **Statements in the upper-right quadrant (the ‘go-zone’). These statements are identified as high-priority for implementation, as indicated by their higher-than-average ratings for ‘ease’ and ‘importance’ to address.**

Focal prompt: *One thing about the lives and experiences of South Asian women that influence their decision, in a positive or negative way, to get screened (i.e., a Pap test or HPV test) for cervical cancer is…*

 

#6 Lack of access to cervical cancer screening information shared by trusted sources

#9 A woman’s lack of understanding and education around cervical cancer

#10 Needing to communicate with healthcare providers in English is a barrier for South Asian women to be screened for cervical cancer

#15 Women need reminders to know when they are due for cervical cancer screening

#18 Not enough media coverage of cervical cancer screening within the South Asian community

#21 Preventative care is not well understood by South Asian women

#41 Women may not know what a Pap test involves

#42 Women may not know the purpose of a Pap test

Box 2 shows us the lower-right quadrant of statements that are considered important to address, but likely challenging to implement. Many of the statements in this quadrant are around fear, stigma, discomfort, family and personal priorities. These are particularly critical to understand because participants found them to be highly important in impacting cervical screening participation for South Asian women, but perceived them as difficult to address. This was discussed extensively during the map interpretation session, as many participants believed that some of the more South Asian culture-specific statements around discussing sexual history and the role of supportive family members really resonated with them. There was also a discussion around the fear in women of finding out they may have precancerous cells or even have cancer, and how that alone can prevent them from engaging with cervical screening. This was emphasized even more when people contextualized this with the many competing work and family priorities in a South Asian woman’s life, and how that could be enough to make them delay or never attend cervical screening. Lastly, the statement around not seeking healthcare unless they are experiencing an issue (statement #3) sparked a discussion around women not feeling the urge to prioritize or seek healthcare if they are asymptomatic and how doing so could become a regrettable decision as they could find out they have a life-altering health issue that they could have found out about early had they attended screening.

Box 2 **Statements in the lower-right quadrant (high importance ratings, low easy to address ratings). These statements are identified as high-priority but challenging to implement.**

Focal prompt: *One thing about the lives and experiences of South Asian women that influence their decision, in a positive or negative way, to get screened (i.e., a Pap test or HPV test) for cervical cancer is…*

 

#3 Women do not go to the doctor unless they are having an issue

#13 Education about cervical cancer is needed for men in South Asian households

#17 South Asian women are not comfortable to discuss their sexual history

#23 South Asian women may be worried about their family finding out they are sexually active

#28 Women may be shy to have an examination in that area of their body

#30 South Asian women may prioritize looking after their families over their own health

#32 Lack of support from family members to go and get screened

#34 Women are afraid to find out if they have cancer

#35 Cervical cancer screening is not openly discussed in the South Asian culture

#44 South Asian women will only get screened when symptoms arise

## 4. Discussion

We engaged multiple South Asian women and different service providers in healthcare services and the community to identify important and feasible actions to address cervical screening participation amongst South Asian women. Participants rated 45 brainstormed statements that composed the master list [29]. Analysis of the rating data that measured participants’ perceptions of the importance of the statements and how easy they would be to address to encourage cervical screening helped us to identify impactful areas of intervention. Overall, we saw that statements were generally rated higher for importance than for being easy to address. The highest rated statements for importance were statements around understanding cervical cancer and Pap tests, shyness to complete a Pap test and needing reminders to know when they are due. Statements that referred to stigma and discomfort around discussing sex were rated as the least easy to address. The go-zone suggested that some of the most impactful areas to address would be around education related to cervical screening, cervical cancer and preventative care. Additionally, this quadrant highlighted that more media coverage in the South Asian community and the trustworthiness of the sources of information are high priorities. Notably, the area below the go-zone—showing us areas of high importance and challenging implementation—has several statements around fear, stigma, norms and social relations. Many of these statements are elements that exist outside of healthcare spaces and are more about family and culture, suggesting that interventions need to take place in the community, but these community interventions may be challenging. For example, one statement in this quadrant was around education for men in households. This statement is further understood when you then consider that other statements in the quadrant are about wanting support from family or being afraid that family may find out someone is sexually active. Educating men in households may be an important point of intervention that can also address the need for support from family and alleviate fear of discussing sexual activity. 

### 4.1. Interventions for Cervical Screening

Our findings suggest that future interventions should be based in the community and focus on effectively communicating and spreading awareness about cervical cancer and screening in a culturally safe way and through trusted sources. Trusted sources can be thought about in many ways. The use of community champions (i.e., known peers) in cervical screening can be particularly effective when they are viewed as being knowledgeable and understanding of the South Asian culture, while also having the time to provide thorough and clear explanations [30]. Work conducted by Wong et al. [31] to increase cervical screening amongst South Asian women in Hong Kong found community health worker-led initiatives to be particularly effective. Strategies that harness the expertise of trained peers to spread awareness and encouragement can also be extended to a woman’s family and friends. 

While findings around education and knowledge issues may not be new to the literature, a new finding from this work is around how to make these knowledge and education strategies more effective. While increasing someone’s knowledge around Pap tests, cervical cancer and preventative care can make a convincing argument for cervical screening, action may be limited by other impactful factors. Statements placed in the area below the go-zone around fear, stigma, norms and social relations that impact the uptake of screening highlight the areas that education and awareness raising must address to be effective and move people from conviction around screening to action. Our study also suggests that while individual education and awareness is needed, efforts to increase cervical cancer screening among South Asians should also include family and friends. The cultural knowledge of peers to recognize attitudinal barriers within households and personal networks may be particularly effective in increasing awareness and addressing stigma around sexual activity. 

Engaging the community will also be important in the design and distribution of educational materials. Chan and So [24] found a community-based multimedia intervention (educational pamphlets and video) to be effective for under- and never-screened South Asian women. A key feature of the intervention was the continuity and sustainability of the efforts that community-based organizations were making to deliver the educational pamphlets and video to clients [24]. This raises an important point about community efforts needing to be sustainable to have a longer impact. 

Additionally, the findings around discomfort with a Pap test and access to screening.

Further support interventions that use HPV self-sampling—a promising method for people who are already underscreenend and/or experiencing various disadvantages [32]. HPV testing is a more accurate form of cervical screening, with many jurisdictions including Ontario beginning to implement it in place of a Pap test. HPV testing uses a vaginal sample that can be clinician- or self-collected. Previous work has shown self-collection to be an acceptable method for some under- or never-screened South Asian women [18,19], with supportive peers playing a critical role in implementation by being engaging, trustworthy sources of information for this new and emerging form of screening [30]. With this upcoming change and what we know about barriers to screening, South Asian women and others who experience similar barriers to a Pap test may greatly benefit from HPV self-sampling. Additionally, peers can also play a role in navigating and attending follow-up procedures for people with abnormal test results as people may similarly be reluctant to attend invasive follow-up care.

### 4.2. Limitations

A main limitation of our study was that people who did not speak conversational English were not included. While needing to communicate with healthcare providers in English came out as a very important barrier to address, we did not include the experiences of people who were unable to effectively communicate in English with their providers. In CM, it would be difficult to maintain the meaning of statements and interpretations across multiple languages. Additionally, our study and identification of areas of intervention did not account for the diversity amongst South Asian women in terms of ethnicity, religion, age, social class, sexual orientation, education and marital status. Future work should focus on identifying the needs of more specific sub-groups.

## 5. Conclusions

We engaged a diverse group of South Asian service users and people who work with and serve them in the community to identify and prioritize areas for intervention to encourage cervical screening amongst South Asian women. Through ratings of importance and being easy to address, we were able to identify impactful and feasible ideas that suggest multiple levels of intervention are needed to create and sustain increased participation in cervical screening. We moved beyond the individual to understand interventions that are needed amongst family, friends, community, healthcare and more. Through concept mapping, we were able to generate new knowledge that is largely participant-driven and that defines the issue of low rates of cervical screening amongst South Asian women as being a result of multiple factors within and outside healthcare. This work highlights key priorities for interventions to better encourage cervical screening. 

## Figures and Tables

**Table 1:** (**a**) Demographic questions asked to all participants before their participation in the brainstorming, rating and map interpretation activities. (**b**) Additional questions asked during the rating and map interpretation activities.

(**a**)					
**Participant Question**	**Options**	**Brainstorming (n = 72)**	**Rating (n = 45)**		**Map Interpretation (n = 9)**
What best describes your role in this study?	I identify as South Asian	52	25		4
	I work in a role or an organization that serves South Asian women AND I identify as South Asian	13	12		4
	I work in a role or an organization that serves South Asian women AND I DO NOT identify as South Asian	1	2		0
	I work as a primary care provider AND I identify as South Asian	2	5		1
	I work as a primary care provider AND I DO NOT identify as South Asian	0	1		0
	Other	4	0		0
If you work in healthcare or in the community, how long have you been in this area of work?	1 to 5 years	5	1		0
	6 to 10 years	3	8		3
	11 to 15 years	4	3		1
	16 to 20 years	2	3		0
	20+ years	4	4		1
Have you ever had a Pap test?	Yes	46	23		9
	No	5	5		0
	Unsure	1	0		0
What is your age?	21 to 30	8	3		1
	31 to 40	19	14		2
	41 to 50	24	19		5
	51 to 60	13	6		1
	61 to 70	8	2		0
Do you identify as	Female	71	45		9
	Male	1	0		0
	Other	0	0		0
(**b**)					
**Participant question**	**Options**	**Rating (n = 45)**		**Map Interpretation (n = 9)**	
If you work in healthcare or in the community, what percentage of the population that you serve are South Asian?		9% to 85%		9% to 85%	
If you work in healthcare or in the community, which of the following describes your role/work? (check all that apply)	Allied health professional (e.g., nurse, physiotherapist, dietician)	4		1	
	Cancer care (screening, diagnosis, treatment)	3		2	
	Community organizer	1		0	
	Community Outreach	3		1	
	Health promoter	4		2	
	Healthcare provider working in a hospital (e.g., hospitalist, inpatient nurse, mammography technician)	3		1	
	Manager	1		0	
	Primary care provider	6		1	
	Program coordinator	4		0	
	Researcher	3		2	
	Settlement services	3		1	
	Volunteer	4		1	

**Table 2:** Average rating values for each statement per rating question. Numbers in far-left column are to identify statements throughout text and figures. Statement numbers do not indicate any value, including rank. Statements in bold indicate high-priority statements identified through go-zone display.

ID	Statement	Average ‘Importance’ Rating	Average ‘Ease’ Rating
1	The belief that you should not “touch” things or go under the knife (meaning any medical procedure) because it brings more harm than good	3.50	2.80
2	Cultural expectations or pressures that the idea of “modesty” prevents women in the South Asian community from getting screened for cervical cancer	3.81	2.86
**3**	**Women do not go to the doctor unless they are having an issue**	4.14	2.80
4	Appointments are not available at times that are convenient for patients	3.70	3.30
5	Women do not feel comfortable with their healthcare provider	3.61	3.09
**6**	**Lack of access to cervical cancer screening information shared by trusted sources**	4.16	3.80
7	Pap test appointments are viewed as time consuming	3.61	3.41
8	Women believing that a Pap test can lead to an infection	3.45	3.79
**9**	**A woman’s lack of understanding and education around cervical cancer**	4.44	3.64
**10**	**Needing to communicate with healthcare providers in English is a barrier for South Asian women to be screened for cervical cancer**	4.00	3.36
11	If a woman believes that cervical cancer is not a severe condition, this can discourage them from getting screened	3.82	3.34
12	Men in South Asian households make decisions about females getting screened	3.36	2.32
**13**	**Education about cervical cancer is needed for men in South Asian households**	4.05	2.75
14	A woman’s belief that cervical cancer screening is not necessary if you have only had one sexual partner	3.70	3.59
**15**	**Women need reminders to know when they are due for cervical cancer screening**	4.30	4.21
16	Negative cultural beliefs behind gynecologist visits leads to South Asian women feeling shame when booking appointments.	3.59	3.00
**17**	**South Asian women are not comfortable to discuss their sexual history**	3.98	2.30
**18**	**Not enough media coverage of cervical cancer screening within the South Asian community**	4.20	3.64
19	Pap tests can feel painful	3.72	3.39
20	Women may view a Pap test as a dirty procedure where you may bleed afterwards	3.52	3.41
**21**	**Preventative care is not well understood by South Asian women**	4.19	3.32
22	Prior negative experience with a Pap test discourages South Asian women from getting screened	3.75	2.77
**23**	**South Asian women may be worried about their family finding out they are sexually active**	4.00	2.73
24	Not having a healthcare provider of a similar cultural background makes intimate tests such as a Pap test, uncomfortable	3.64	2.91
25	Sex is a taboo topic amongst South Asians	3.75	2.39
26	Any tests related to sex can be considered dirty	3.70	2.66
27	Women believe that if they have an HPV vaccine, they do not need to be screened for cervical cancer	3.39	3.72
**28**	**Women may be shy to have an examination in that area of their body**	4.23	2.73
29	Foreign trained physicians may not encourage their patients to do cancer screening, as preventative care may not have been common in their home countries.	3.50	3.45
**30**	**South Asian women may prioritize looking after their families over their own health**	4.14	2.72
31	South Asian women may be too busy with their jobs or careers to take care of their own health	3.77	2.77
**32**	**Lack of support from family members to go and get screened**	3.86	2.68
33	Lack of support from friends to go and get screened	3.60	3.16
**34**	**Women are afraid to find out if they have cancer**	3.98	2.70
**35**	**Cervical cancer screening is not openly discussed in the South Asian culture**	4.14	2.89
36	Women may be uncomfortable with going to the doctor in general	3.82	2.77
37	Women hear other women share negative experiences about getting a Pap test	3.59	2.98
38	The belief that if a cervical cancer diagnosis is your fate or destiny, there is no reason to get screened	3.32	3.07
39	Belief that you only have to worry about cervical cancer if you have a problem with your menstruation	3.61	3.66
40	Family doctor does not encourage cervical cancer screening during appointment	3.64	3.73
**41**	**Women may not know what a Pap test involves**	4.25	4.02
**42**	**Women may not know the purpose of a Pap test**	4.25	3.95
43	Women do not have a family doctor	3.68	3.16
**44**	**South Asian women will only get screened when symptoms arise**	4.20	2.86
45	South Asian women won’t get screened because they think they cannot get cervical cancer	3.41	3.23

## Data Availability

The datasets generated and/or analyzed during the current study are not publicly available due to maintaining the privacy and confidentiality of participants, but are available from the corresponding author on reasonable request.
